# Knowing Your Audience: A Typology of Smoke Sense Participants to Inform Wildfire Smoke Health Risk Communication

**DOI:** 10.3389/fpubh.2020.00143

**Published:** 2020-05-05

**Authors:** Mary Clare Hano, Steven E. Prince, Linda Wei, Bryan J. Hubbell, Ana G. Rappold

**Affiliations:** Office of Research and Development, United States Environmental Protection Agency, Durham, NC, United States

**Keywords:** wildfire, smoke sense, smoke, citizen science, risk communication, mobile application

## Abstract

Central to public health risk communication is understanding the perspectives and shared values among individuals who need the information. Using the responses from a Smoke Sense citizen science project, we examined perspectives on the issue of wildfire smoke as a health risk in relation to an individual's preparedness to adopt recommended health behaviors. The Smoke Sense smartphone application provides wildfire-related health risk resources and invites participants to record their perspectives on the issue of wildfire smoke. Within the app, participants can explore current and forecasted daily air quality, maps of fire locations, satellite images of smoke plumes, and learn about health consequences of wildfire smoke. We used cluster analysis to identify perspective trait-clusters based on health status, experience with fire smoke, risk perception, self-efficacy, access to exposure-reducing resources, health information needs, and openness to health risk messaging. Differences between traits were examined based on demographics, health status, activity level and engagement with the app. We mapped these traits to the Precaution Adoption Process Model (PAPM) to indicate where each trait lies in adopting recommended health behaviors. Finally, we suggest messaging strategies that may be suitable for each trait. We determined five distinct perspective traits which included individuals who were *Protectors* and have decided to engage on the issue by adopting new behaviors to protect their health; *Cautious, Proactive, and Susceptible* individuals who were at a *Deciding* stage but differed based on risk perceptions and information needs; and the *Unengaged* who did not perceive smoke as a health issue and were unlikely to change behavior in response to messaging. Across all five traits, the level of engagement and information needs differed substantially, but were not defined by demographics. Individuals in the *Susceptible* trait had the highest level of engagement and the highest information needs. Messaging that emphasizes self-efficacy and benefits of reducing exposure may be effective in motivating individuals from the deciding stage to taking health protective action. Shared perspectives define an individual's propensity for acting on recommended health behaviors, therefore, health risk message content should be tailored based on these perspectives.

## Introduction

In the United States and across the world, large-scale wildfires are occurring more frequently, and with these fires comes smoke (1). Smoke from wildfires is made up of air pollutants which have been associated with a range of negative health outcomes such as cardiovascular and respiratory diseases and illnesses, reproductive effects, and premature death (2, 3). In response to these outcomes and to prepare for smoke intrusion, many communities are developing health risk communication strategies, but little is known about how effective current strategies are at communicating what is known and motivating action to reduce outcomes (4–6). Theory and conventional practice related to public health communication suggests the content and context of messaging are important to effectiveness, and different contexts may require different content (7, 8). Intended audiences may be more likely to understand and act upon messaging that takes into consideration their lived experiences and their social and environmental context (7–12).

While public health guidelines for risk communication recommend becoming familiar with the intended audience (13–15), that can be a challenging endeavor in the context of wildfire smoke given this audience may encompass entire communities or regions with diverse populations representing a range of experiences and perspectives. Further, challenges related to crafting messages may be compounded by the need to incorporate complex information about wildfires, smoke, air quality, known health risks of exposure, and effectiveness of recommended exposure-reducing behaviors (16). Little research has been conducted on the contexts that may influence health risk communication strategies about wildfire smoke.

This study explores perspectives on wildfire smoke as a health risk among participants of Smoke Sense—a citizen science project with an objective to engage affected individuals on the issue and to inform effective health risk communication strategies which motivate individual level behavior change. The Smoke Sense mobile phone app provides wildfire related resources, otherwise available through disparate sources (NWS, AirNow, Smoke Outlook, etc.), together with health risk messaging. Participants can explore current and forecast air quality, learn about the status of wildfires, visualize the extent of smoke exposure, learn about what air quality means to their health, and get tips on how to protect their health. Participants are also invited to report their observations of smoke, current health symptoms, and health behaviors taken in response to smoke (17, 18). Findings from the pilot season, August 1, 2017 through January 7, 2018, indicated a strong demand for personally relevant data during wildfire smoke events (17). Rappold (17) reported that most of the users clearly recognized smoke as a health risk and as an environmental hazard. During the pilot season, over 80% of user-reported smoke observations matched satellite-based measures of smoke. However, users engaged in health protective behaviors in response to symptoms rather than as preventative measures, which is the ultimate goal of engagement with a health risk messaging. This suggests there is an opportunity for improving health risk communication that will more effectively prompt preventative action.

App-based communication platforms can provide direct messaging to individuals who are experiencing smoke when and where they need it. However, little is known about how personal context such as basic risk perception, previous experience with the issue, and self-efficacy, motivate app users to adopt recommended health behaviors. Yet, understanding the intended audience and their personal context is critical to developing of health risk messages that are likely to motivate progression along the stages of change and adoption of recommended health behaviors (19).

Smoke Sense provides a unique opportunity to gain collective knowledge about the role of personal context among individuals who have access to the same information content during a wildfire smoke episode. Here we explored individuals' perspectives on the issue of wildfire smoke as a health risk to understand whether there is heterogeneity of perspectives among participants or a similar overall, global view. We examined whether perspectives and engagement with the app are determined by individual demographic characteristics and current health conditions. Finally, we explored the theories of change that may be applicable to inform development of effective messages, and suggest messaging strategies that may be effective in moving users toward health protective behavior.

## Methods

The Smoke Sense app is free to download and is available on both iOS and Android operating systems. Participation in the Smoke Sense project through the app is open to anyone in the United States over 18 years old who has a smartphone. Over 30,000 individuals have downloaded Smoke Sense since it launched in late summer 2017. The present study uses individual-level data from the Smoke Sense citizen science project reported between September 2018 and May 2019, which included some of the most severe smoke episodes in recent history (20). Individuals engage directly with the app to the extent that they wish, and there are no required components or questions beyond entering a ZIP code of interest, which is used as a location anchor for the displayed air quality information (18, 21). All users are anonymous and no personally identifiable information is collected. Responses are transmitted through an API to a database located on an EPA-owned and administered server from where they are retrieved for analysis. The questions include response-option formats such as Likert-type, multiple choice, and categorical options. For analysis, questions with categorical responses are coded as dummy variables and those with Likert-type responses as ordered values. All data used in this study are available for further investigation through U.S. EPA ScienceHub site.

Once an individual downloads the app onto their phone, they have access to the four app modules including: (1) Fire and Smoke Near Me, which is an air quality information module providing maps of satellite-detected fires and smoke as well as current and forecast air quality reports; (2) the Air Quality Index (AQI), which translates what air quality means to the participant's health; (3) the AQ101 module, which is an interactive health risk messaging quiz module; and (4) the Symptoms & Smoke Observations module, where participants can report observations of smoke and concurrent health symptoms. In-app badges are awarded to individuals for engaging with these modules. Participants were awarded the *User Badge* for interacting with the AQI component once per week, and an *Explorer Badge* for viewing the maps in the Fire and Smoke Near Me module once per week. The *User* and *Explorer Badges* represent information seeking interaction on behalf of the participant, in that participants were awarded these badges for seeking information about the air quality. The *Observer* and *Learner Badges* were awarded for deeper engagement through reporting experiences and interacting with the health risk messaging components of the Smoke Sense app. When a participant made an entry in the weekly Symptoms & Smoke Observations module, they received an *Observer Badge* and when they interacted with the health risk messaging quiz module, AQ 101, they received a *Learner Badge*.

### Data

We draw on two types of Smoke Sense data in the present study. The first are responses in the My Profile section, which included basic demographics, health status, activity patterns, and perspectives on wildfire smoke. The second type of data are engagement metrics based on in-app badges awarded to users for interacting with app modules. The perspectives were measured based on profile questions about: personal assessment of health; access to exposure-reducing resources, e.g., air purifier, whole house air conditioning, air quality warnings, etc.; reported frequency of smoke where they live; perspectives on health risks of smoke exposure; self-efficacy for reducing exposure; perspectives on usefulness of information alerts; information needs about smoke and health; and frequency of health symptoms. Demographic data included gender, age group, race/ethnicity, education level, current health conditions, and level of outdoor activity. The exact format of question items with response options are provided in Supplemental Materials Table 1, while distribution of demographic data is provided in Table 1. We used Smoke Sense badge data to characterize the level of engagement and participation in the app.

**Table 1:** Summary Statistics of Smoke Sense Participants Between September 2018 and May 2019.

	**Number of responses**	**Percent of responses (%)**
**Sex (*****n*** **=** **5,018)**		
Female	2,647	52.75
Male	2,274	45.32
Other	81	1.61
N/A	16	0.32
**Age (*****n*** **=** **5,018)**		
18–29	747	14.89
30–39	1,090	21.72
40–49	1,131	22.54
50–64	1,399	27.96
65+	637	12.69
N/A	14	0.28
**Race/ethnicity (*****n*** **=** 5,018)^*^		
African-American/Black	109	2.17
Asian/Pacific Islander	435	8.67
Hispanic/Latino	393	7.83
Native American	90	1.79
White	3,829	76.31
Other	354	7.05
N/A	46	0.92
**Education (*****n*** **=** **5,018)**		
High school degree, GED, or less	633	12.61
Technical school, trade or vocational training	808	16.10
College, masters, doctorate, or professional degree	3,487	69.49
N/A	90	1.79
**Average outdoor activity (*****n*** **=** **5,018)**		
Not very active	242	4.82
Mild (walking, standing)	2,239	44.62
Moderate (regular jog, gardening)	1,661	33.10
Very Active (run, bike daily, work outdoors)	876	17.46
**Currently health conditions (*****n*** **=** 5,018)^*^		
Asthma	980	19.91
Chronic obstructive pulmonary disease (COPD)	96	1.95
Other respiratory disease	165	3.35
Hypertension or high blood pressure	629	12.78
Other heart disease	145	2.95
Type II diabetes, metabolic syndrome, or obesity	315	6.40
Allergies related to the upper respiratory tract, eyes, and ears	1,361	27.65
Other chronic disease	403	8.19
None of the above	2,383	48.42

Smoke Sense participant summary demographics. Questions are from data collected through September 2018 through May 2019. Minor additive discrepancies are due to rounding.

* *Survey questions regarding race/ethnicity & current health conditions allowed for multiple categories to be marked; total and percentage are greater than total n 100%*.

### Analysis

The research questions that guide this study are:

RQ1: Do individuals tend to share a global perspective on the issue of wildfire smoke and health? Or is there heterogeneity of perspectives?
RQ2: Are perspectives and engagement with information determined by demographics and health status?
RQ3: Do these perspectives map to existing theories of individual-level health behavior change?

To address RQ1 we performed cluster analysis on the measured perspectives from participant responses. More specifically, we used agglomerative hierarchical complete-linkage clustering (21–23) which is a data reduction technique that identifies clusters based on proximity of response units to one another, one by one, in a series of iterations (21, 22, 24). We used a complete linkage method, where the distance between clusters was the furthest distance between pairs of cases in each cluster.

A Gower distance matrix was generated using the daisy() function from the cluster v2.1.0 package in R 3.6.1 to compute all pairwise dissimilarities between cases in the data set. The hclust() function from the stats v3.6.1 package in R was then used to perform agglomerative hierarchical clustering using complete linkage clustering. Model validation metrics produced for each clustering solution reflected the similarity of cases within a cluster (e.g., root-mean-square standard deviation) and the uniqueness from different clusters (e.g., R-squared and the Modified Hubert Γ statistic) (25). Comparison of within-trait sum of squares across clustering solutions is used to help determine the appropriate number of clusters within the dataset (Supplemental Material Figure 1).

To address RQ2 we used a multinomial logit model to estimate the probability that a Smoke Sense participant will be classified into a given perspective trait as a function of age, gender, race, education, and average self-reported outdoor activity level. This information tells us whether specific demographics are more likely to share common perspective traits.

Smoke Sense participants could engage at four levels, as Users, Explorers, Observers, and Learners. The first two levels are mainly information seeking behaviors, while active reporting of observations and learning on the issue are deeper levels of engagement. To examine the level of engagement based on each badge type among individuals in different trait-clusters we used one-way analysis of variance.

To address RQ3 we looked to individual-level theories of health behavior change to understand how existing theory can provide a framework for application of these findings in risk communication strategies related to wildfire smoke and health. Specifically, we positioned the perspective traits across the stages of behavior adoption process theories. Finally, we recommend the type of message content that would help move individuals in each trait along the stages of change.

## Results

During the study period (September 2018 and May 2019), a total of 5,018 Smoke Sense participants provided responses to questions in the My Profile section related to their perspectives on smoke as a health risk. Demographic summary statistics for these 5,018 individuals are shown Table 1. Individuals in this sample are predominantly white (76.31%) and college educated (69.49%). A slight majority are female (52.75%) and the majority are middle age between 30 and 65 years old (72.22%).

### Smoke Sense Participant's Perspectives on Wildfire Smoke as a Health Risk

Overall, individuals in this sample reported good to excellent health (92%), and 43% reported experiencing no health symptoms (Table 2). Smoke was common where they lived with almost three-quarters (73%) indicating agreement. There was generally high agreement that wildfire smoke is a health risk and that exposure can be reduced, indicating a perception that smoke is a modifiable health risk. Among the list of exposure-reducing resources listed in the user profile question, participants had the greatest access to a vehicle with recirculate mode, whole house air conditioning, and the AQI. However, there was variation between information needs, with an approximately equal percent of individuals preferring: information on specific measures to protect health; additional information on how it impacts health; information from trusted sources (verifiable); and no additional information on the subject.

**Table 2:** Summary Sample Statistics on Measures of Perspectives Related to Wildfire Smoke as a Health Risk.

	***N***	**%**
**Personal health assessment:** ***n*** **=** **5,018** “Would you say your own health, in general is:”		
Excellent	1,188	23.67
Very good	2,124	42.33
Good	1,318	26.27
Fair	318	6.34
Poor	70	1.39
**Personal health current symptoms:** ***n*** **=** **5,018** “Do you commonly experience any of these symptoms? (Select all that apply):”		
Coughing, trouble breathing, wheezing, asthma attacks, or similar	918	18.29
High blood pressure, chest pain or tightness, rapid or irregular heartbeat, or similar	428	8.53
Stinging eyes, scratchy throat, or similar	1,156	23.04
Runny or stuffy nose, irritated sinuses, or similar	1,766	35.19
Tiredness, headaches, or similar	1,459	29.08
None of the above	2,167	43.18
**Experience with wildfire smoke:** ***n*** **=** **5018** “Smoke from wildfires is a common occurrence where I live.”		
Strongly agree	1,898	37.82
Somewhat agree	1,763	35.13
Neither agree nor disagree	620	12.36
Somewhat disagree	364	7.25
Strongly disagree	373	7.43
**Risk perception:** ***n*** **=** **5,018** “A few hours of wildfire smoke in the air can impact my health.”		
Strongly agree	2,805	55.90
Somewhat agree	1,416	28.22
Neither agree nor disagree	567	11.30
Somewhat disagree	142	2.83
Strongly disagree	88	1.75
**Self-efficacy to reduce exposure:** ***n*** **=** **5,018** “It is possible for me to reduce my wildfire smoke exposure.”		
Strongly agree	1,632	32.52
Somewhat agree	2,132	42.49
Neither agree nor disagree	757	15.09
Somewhat disagree	339	6.76
Strongly disagree	158	3.15
**Access to exposure-reducing resources:** ***n*** **=** **5,018** “Which of the following do you use or have readily available to use? (Select all that apply)”		
A HEPA (high efficiency particulate air) room purifier	1,468	29.25
A car with recirculate mode for the ventilation system	2,890	57.59
Single room air conditioner(s)	616	12.28
Whole house (central) air conditioning	2,358	46.99
Workplace air conditioning	1,801	35.89
An N95 (or similar) respirator mask	1,876	37.39
Protective gear e.g. plastic gloves and goggles	909	18.11
Access to AQI and AQ health related warnings	2,114	42.13
None of the above	559	11.14
**Health messaging receptiveness: n = 5,018** “Information alerts are likely to help me reduce my exposure to wildfire smoke.”		
Strongly agree	2,739	54.58
Somewhat agree	1,594	31.77
Neither agree nor disagree	522	10.40
Somewhat disagree	96	1.91
Strongly disagree	67	1.34
**Information needs:** ***n*** **=** **5,018** “Before considering reducing wildfire smoke exposure, I need more information on: (Select all that apply)”		
Whether smoke impacts my health	1,404	30.59
Whether specific measures will help my health	1,818	39.61
Whether the measure was recommended by a trusted source	1,702	37.08
The effort required for a specific measure	1,098	23.92
The monetary costs of a specific measure	898	19.56
I would not consider reducing exposure	86	1.87
I don't need additional information before I reduce exposure	1,617	35.23

Cluster analysis classified individuals' perspectives on smoke as a health issue into three, four, and five distinct cluster trait solutions (see Supplemental Material). Beginning with the three-cluster solution, the cluster defining variables were health, risk perception, access to resources, self-efficacy for reducing exposure, receptiveness to health messaging, and information needs. In the four-cluster solution, one of the initial three clusters split into two, on the variables: health, access to exposure-reducing resources, and risk perception. In the five-cluster solution, another cluster split into two based on health and information needs. The five-cluster solution accounted for the largest portion of variation and in greater than five-cluster solutions, the magnitude in explanatory power indicated by decreases in within cluster sum of squares begins to taper off (Supplemental Material Figure 1). Described here are the results from the five-cluster solution, with a descriptive label provided to facilitate disambiguation for each resulting trait. Descriptive labels are intended to characterize the relationship with health, air quality, and exposure-reducing behaviors among individuals attributed to that trait. A summary of these traits is shown in Table 3.

**Table 3:** Perspective traits cluster by measures of health status, risk perception, self-efficacy, openness to health risk messaging, health information needs, and access to exposure reducing resources.

		Health: “Would you say your own health, in general is:”^*^ “Do you commonly experience any of these symptoms? (Select all that apply):”^**^		
		Poor	Good	Excellent
Risk perception: “A few hours of wildfire smoke in the air can impact my health.”^***^	High	Protectors, susceptible	Cautious	
	Medium			Proactive
	Low			Unengaged
Self-efficacy: “It is possible for me to reduce my wildfire smoke exposure.”^***^	High		Cautious	Proactive
	Medium	Protectors, Susceptible		
	Low			Unengaged
Receptiveness: Health risk communication: “Information alerts are likely to help me reduce my exposure to wildfire smoke.”^***^	High	Susceptible	Cautious	Proactive
	Medium			
	Low	Protectors		Unengaged
Information needs: “before considering reducing wildfire smoke exposure, I need more information on: (select all that apply)”^∇^	High	Susceptible	Cautious	
	Medium			Proactive
	Low	Protectors		Unengaged
Access: exposure-reducing resources: “which of the following do you use or have readily available to use? (select all that apply)”^⊗^	High		Cautious	Proactive
	Medium	Protectors, susceptible		
	Low			Unengaged
Experience with wildfire smoke: “Smoke from wildfires is a common occurrence where I live.”^***^	High	Protectors, susceptible	Cautious	
	Medium			Proactive
	Low			Unengaged

Health status was the most influential factor.

* : 1) Excellent; 2) Very Good; 3) Good; 4) Fair; 5) Poor.

** : 1) Coughing, trouble breathing, wheezing, asthma attacks, or similar; 2) High blood pressure, chest pain or tightness, rapid or irregular heartbeat, or similar; 3) Stinging eyes, scratchy throat, or similar; 4) Runny or stuffy nose, irritated sinuses, or similar; 5) Tiredness, headaches, or similar; 6) None of the above.

*** : 1) Strongly agree; 2) Somewhat agree; 3) Neither agree nor disagree; 4) Somewhat disagree; 5) Strongly disagree.

∇ : 1) Whether smoke impacts my health; 2) Whether specific measures will help my health; 3) Whether the measure was recommended by a trusted source; 4) The effort required for a specific measure; 5) The monetary costs of a specific measure; 6) I would not consider reducing exposure; 7) I don't need additional information before I reduce exposure.

⊗ *: 1) A HEPA (high efficiency particulate air) room purifier; 2) A car with recirculate mode for the ventilation system; 3) Single room air conditioner(s); 4) Whole house (central) air conditioning; 5) Work place air conditioning; 6) An N95 (or similar) respirator mask; 7) Protective gear, e.g., plastic gloves and goggles; 8)Access to AQI and AQ health-related warnings; 9) None of the above*.

The first trait in the five-cluster solution, the health *Protectors*, included 1,197 individuals (23.9 %) who reported low overall health and a variety of conditions affecting their health at the time. These individuals reported that smoke from wildfires is common where they live and agreed that smoke is a risk to their health. They reported moderate self-efficacy for reducing their exposure and have access to a variety of resources to help reduce exposure. Compared to other traits, the Protectors report very little need for additional information, with 68% stating they don't need any additional information before deciding to reduce their exposure. This trait is higher percentage female, reports higher than sample average health conditions, lower than average outdoor activity levels, and lower than average post-secondary education. Detailed breakdown of demographic characteristics by cluster trait are given in the Supplemental Material Table 3 and Supplemental Material Figures 2–7.

The second trait in the five-cluster solution, the *Cautious*, included 1293 individuals (25.8%). This trait was in the middle with respect to self-reported health compared to other traits. Individuals in this trait reported the strongest agreement that smoke is common where they live and strongly agreed that smoke impacts health. They reported high self-efficacy and had higher than sample average access to exposure-reducing resources such as air conditioning, air purifiers, and respirators. They reported having high information needs about smoke, health risks, and strategies before considering reducing their exposures. The Cautious trait was predominantly white, middle-age, female, with high levels of post-secondary education. They reported mild outdoor activity and higher than average levels of asthma and allergies.

The third trait, the *Proactive*, included 1,421 individuals (28.3%). This trait was healthier compared to the overall sample, and they reported almost no current health conditions. They reported smoke is common where they live, and overall agreement that smoke affects their health. They also report high self-efficacy for reducing their exposure and have high access to a variety of exposure-reducing resources. They tend to view information alerts as helpful and have average information needs about specific exposure-reducing strategies and related costs/effort. The Proactive trait is mostly white, middle-age, men. They reported fewer than average current health conditions (73% reporting none), and higher than average outdoor activity levels. This trait contains the highest percentage of post-secondary education at 80%.

The fourth trait, the *Susceptible*, includes 502 individuals (10.0%). This trait reports the poorest general health and the most symptoms. They have the strongest agreement that smoke impacts health and moderate self-efficacy for reducing exposure. They generally agree that information alerts are useful to help reduce their exposure and have high information needs about smoke risks and strategies. They are well above the sample average for all health conditions, and they report below-average outdoor activity levels. The Susceptible trait is predominantly female and has the lowest percentage of male individuals across all traits.

There were 605 individuals (12.1%) included in the fifth trait, the *Unengaged*. Individuals in this trait were in very good health and report almost no current health symptoms. Compared to other traits, they report smoke as being less common where they live. They had below average perspectives of smoke as a health risk, with lower than average self-efficacy for reducing their exposure. The Unengaged had the highest percentages of people being undecided about the helpfulness of information alerts about smoke and reported relatively low needs for additional information. Notably, they reported the least access to any resources to help reduce their exposure, with over half indicating they had no access to any resource listed. They had the lowest percentage of individuals reporting race/ethnicity as White, although still the majority at 69%. Ten percentage of individuals reported race/ethnicity as Hispanic/Latino, and 12% reported Other. Sixty percentage indicated they were male, and 22% were in the 18–29 age range, well above the sample mean of 15%. The Unengaged trait also contained the highest percentage of high school degree or less, and compared to others, this trait had the lowest percentage of post-secondary education (though still the majority).

### Are an Individual's Demographic Characteristics Associated With Perspectives of Smoke as a Health Risk?

This analysis examined the relationship between a participant's demographic variables and cluster trait membership. The variables included in this analysis included participant gender, age, race, education, activity level, and current health conditions. Although distribution of demographics differed by cluster trait, demographic characteristics were not a major factor in defining perspectives on the issue. These data are included in Supplemental Material Table 3.

Male participants were most likely to be attributed to the Proactive trait, whereas female participants had approximately equal odds of being attributed to the Protector and Cautious traits. Individuals who are 18–29 had similar odds of being attributed to the Protectors, Cautious, or Proactive traits. Respondents in the 30–39, 40–49, and 50–64 age ranges had the highest probabilities of being attributed to the Proactive trait, while those who are 65 and older had the highest odds of being attributed to the Protectors trait. Participants who report race/ethnicity as: African American, Asian/Pacific Islander, Hispanic/Latino, or Native American have higher odds of being attributed to the Cautious trait, while those who are White have a greater probability of being attributed to the Proactive trait. Individuals who reported a high school degree, GED, or less have the highest odds of being attributed to the Protectors trait; those with a technical, vocational, or associate degree level into the Cautious trait; and those with a bachelor or graduate degree into the Proactive trait. Finally, individuals who reported no current health conditions were most likely to be attributed to the Proactive trait. Those most likely to be attributed to the Protectors trait include respondents who report respiratory conditions including asthma, allergies, and chronic obstructive pulmonary disease (COPD) as well as other heart disease. Individuals who report hypertension; Type II diabetes, metabolic syndrome, or obesity; or other respiratory or chronic disease had the highest probability of being attributed to the Cautious trait. People who reported *any* current health condition had the lowest odds of being attributed to the Unengaged trait. The response variable with the highest probability of predicting cluster trait was reporting “no current health conditions.”

### Is There a Relationship Between Perspectives About Wildfire Smoke as a Health Risk and the Engagement With Smoke Sense?

Across clusters, individuals tended to engage with the Smoke Sense app differently. Defining engagement as interaction with the app modules, engagement is measured with badges that a participant accrues. Badges are awarded to participants based on their interaction with the app modules. There were significant differences in mean number of badges across traits for both overall engagement (total badges) and for specific types of engagement, as measured by specific badge types. There were no significant differences across traits for badges awarded based on information seeking engagement (Profile, User, and Explorer badges), which indicated that individuals across traits are interacting with the app similarly when receiving information (air quality, smoke, etc). However, there were significant differences in how different traits engaged with the app at a deeper level (Observer and Learner badges). Notably, the Susceptible trait, followed by Cautious trait, had the highest engagement as Observers and Learners. Participants in the Unengaged trait had the lowest levels of engagement overall and in each of the four categories (Users, Explorers, Observers and Learners). Table 4 shows the mean number of badges by cluster trait as well as the F-ratio for comparisons across traits by specific badge type.

**Table 4:** Summary Statistics of Smoke Sense Participant Engagement.

**Badge type^**#**^**	**Mean number of badges (SD)**					**ANOVA**^**+**^ **F-ratio and significance**
	**Protectors *n* = 1,136**	**Cautious *n* = 1,246**	**Proactive *n* = 1,356**	**Susceptible *n* = 477**	**Unengaged *n* = 567**	
User	4.71 (5.40)	4.68 (4.98)	4.48 (5.16)	4.70 (5.26)	4.04 (4.36)	F (4, 4,777) = 2.049
Explorer	4.36 (5.20)	4.21 (5.13)	4.32 (5.03)	4.38 (4.74)	3.95 (4.31)	F (4, 4,777) = 0.839
Observer	0.97 (2.41)	1.09 (2.35)	1.07 (2.53)	1.32 (2.86)	0.53 (1.72)	F (4, 4,777) = 8.242^***^
Learner	1.40 (2.28)	1.59 (3.39)	1.39 (3.03)	2.01 (4.36)	0.86 (2.16)	F (4, 4,777) = 9.587^***^

Smoke Sense participants could engage at five levels as Users, Explorers, Observers, and Learners earning respective badges. Profile badge is awarded once per participation for completing a full profile while others are awarded once a week. The first three levels reflect information seeking behaviors, while active reporting of observations and learning on the issue reflect deeper levels of engagement. To examine the level of engagement with the app among individuals in different trait-clusters for each level of engagement, we used one-way analysis of variance.

*** statistically significant at.005 level.

# User, Explorer, Observer, and Learner badges are awarded once per week. The values in the table indicate average number of weeks participants engaged with respective modules.

+ *One way analysis of variance was done for each badge type*.

### Do Perspective Traits Map to Existing Theories of Individual-Level Health Behavior Change?

#### Protectors: Decided to Act

**Figure 1:**
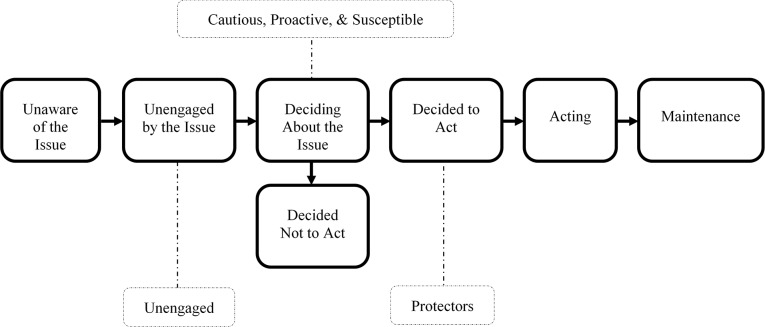
Perspective traits position on stages of decision making.

The Precaution Adoption Process Model is one model that we evaluated as appropriate for understanding the perspective traits, and we have mapped each trait to the model as shown in Figure 1 (26, 27). We positioned the Protectors trait in the “Decided to Act” stage of the Precaution Adoption Process Model due to their high agreement that smoke is a health risk and high readiness to act to reduce that risk without needing additional information. Considering these factors collectively, we propose these individuals are likely concerned with health and are motivated to reduce risks that may jeopardize their health status. This trait's profile and engagement levels suggest these individuals are seeking cues about air quality and smoke that tell them *when* to act, rather than seeking educational messaging that tells them *why* or *how* to act. Perspective and engagement profiles common to this trait suggest these individuals are decided and ready to act. A summary of findings is shown in Table 5.

**Table 5:** Summary of findings by perspective trait.

		**Protector**	**Cautious**	**Proactive**	**Susceptible**	**Unengaged**
Measures of perspectives	Health	Poor	Good	Excellent	Poor	Excellent
	Risk perception	High	High	Medium	High	Low
	Self-efficacy	Medium	High	High	Medium	Low
	Receptiveness to health risk communication	Low	High	High	High	Low
	Information needs	Low	High	Medium	High	Low
	Access to exposure reducing resources	Medium	High	High	Medium	Low
	Experience with smoke	High	High	Medium	High	Low
Engagement by smoke sense app module type	Information modules	High	High	Medium	High	Low
	Reporting modules	Medium	Medium	Medium	High	Low
	Educational module	Medium	Medium	Medium	High	Low
Major demographic probabilities	Race/ethnicity	White or Latino	Non-White	White	Native American	Other
	Gender	Female	Female	Male	Female	Male
	Age	Approx. equal probability across age groups	Approx. equal probability across age groups	30–49	18–29 or 65+ than 30–64	18–29
	Education	High school or vocational	Vocational	Post-secondary	High school or vocational	High school
	Average outdoor activity	Low to moderate	Low to moderate	Moderate to high	Low	Moderate to high
	Current health conditions	Allergies, asthma, chronic obstructive pulmonary disease, other chronic disease	Allergies, asthma, hypertension, diabetes, other chronic disease	Low probability across conditions	Low to moderate probability across conditions	Low probability across conditions
Location on precaution adoption process model		Decided to act	Deciding about the Issue	Deciding about the Issue	Deciding about the Issue	Unengaged by the Issue
Propositions for health risk messaging		Underscore self-efficacy for reducing exposure and nudge toward action	Link exposure with subclinical outcomes	Emphasize exposure as risk to maintaining well-being	Contextualize exposure as a modifiable risk	Underscore impact of smoke on health and activities

*The table summarizes the demographics and engagement among the five observed perspective traits on the issue of wildfire smoke as a health risk (Protector, Cautious, Proactive, Susceptible, and Unengaged). Perspective traits were mapped at the different stages of the Precaution Adoption Process Model*.

These individuals will likely benefit from health-based risk communication messaging that includes aspects to enhance self-efficacy for reducing exposure and provides a nudge toward action. People with diseases associated with the lungs, including asthma, allergies, and COPD, are most likely to be attributed to the Protector trait. Interpretation of the cluster analysis suggests individuals in this trait have higher estimates of risk perception but a medium level of self-efficacy. These individuals are likely to have decided to act and may benefit from risk communication that incorporates self-efficacy messaging. In addition to health risk messaging through mobile apps, communicating through physicians, including internists, immunologists, and pulmonologists may be an effective strategy to reach these individuals who are at increased risk and may be ready to act.

#### Cautious, Proactive, Susceptible: Deciding About the Issue

We positioned Cautious, Proactive, and Susceptible traits in the “Deciding to Act” stage of the Precaution Adoption Process Model framework. Individuals with these traits will likely benefit from messaging that homes in on their high informational needs and issues related to their personal concerns. The Cautious trait will likely benefit from health education messaging that links exposure to symptoms that individuals may experience. Contextualizing sub-clinical symptoms, such as stinging, watery eyes and coughing as a function of wildfire smoke exposure may help these individuals make the connection between exposure and regularly experienced symptoms they report in My Profile. Characterizing the cost-benefit relationship between their exposure and potentially avoiding these symptoms may be especially salient. Individuals within the Proactive trait reported being in the best health and will likely benefit from messaging that emphasizes smoke exposure as a risk to maintaining well-being that may impact outdoor recreation opportunities. Those grouped within the Susceptible trait will likely benefit from health education messaging that contextualizes smoke exposure as modifiable. These individuals will also likely benefit from messaging that includes self-efficacy components and emphasizes that the benefits of reducing exposure may exceed the costs (e.g., financial, time, and effort).

#### The Unengaged: Unengaged by the Issue

We placed the Unengaged in the “Unengaged by the Issue” stage of the Precaution Adoption Process Model, largely because these individuals report low levels of risk perception, low self-efficacy, little access to resources that can reduce their exposure, and low receptiveness to health information about smoke. The perspective profile for this trait suggests these individuals may be far from adopting recommended behaviors. These individuals report being in excellent health with almost no symptoms, they are younger, very active, mostly male, and while the majority report post-secondary education, 20% report a high school degree or less, which indicates a portion of individuals in this trait may be early career or current post-secondary/vocational students.

In terms of interaction with the app, the Unengaged have the lowest mean number of badges for all badge types across traits. Their engagement was mostly information seeking and not with the health risk communication features of Smoke Sense (learning, reporting). Interestingly, approximately 64% of individuals in this trait reported “I don't need any additional information before acting.” This is the second highest percentage of all traits, following the Protectors, which we placed on the opposite end of the model from Unengaged. However, when considering the lowest level of risk perception in this group, we expect that these individuals would consider reducing exposure if they believed it to be relevant to them personally. These individuals probably do not make a connection between exposure and their health because of their exceptionally good health and young age. It is likely that, as many people do, these individuals believe that effects they are experiencing are mild, temporary and reversable. Highlighting the factors that increase susceptibility (e.g., asthma, heart condition) is not likely to resonate with this group. Instead, messages that emphasize the impact that exposure may have on what these individuals already care about (e.g., good health and fitness) and emphasizing actions they already do (e.g., exercise) may be more salient. Messaging related to motivations for the high levels of outdoor activity reported in this trait may be particularly salient (e.g., reducing exposure is good for the same reasons that exercise is).

## Discussion

Engagement with the Smoke Sense citizen science project reflects how individuals interact with and process information about air quality, wildfires, and health risks through an app-based health risk communication resource. In this study we examine individuals' perspectives on wildfire smoke as a health risk and the role of those perspectives on engagement in the Smoke Sense project. Most participants reported being in good to excellent health, identified smoke as a health risk, had previous experience with wildfire smoke, and were open to health risk messaging. We identified five distinct traits based on their perspectives and attributes, which varied on the measure of overall health, levels of risk perception, access to exposure-reducing resources, information needs, self-efficacy, and receptiveness to health risk communication. While these traits were not strongly identified by demographics, individual engagement with this health risk communication tool differed across traits.

Health behavior stages of change models, such as the Transtheoretical Model, or Precaution Adoption Process Model, provide a framework for understanding an individual's general progression toward adopting recommended health behaviors, which are usually communicated through risk communication messaging (11). Taking into consideration each trait's full perspective profile, we map the traits identified in this analysis onto the Precaution Adoption Process Model shown in Table 5 (adapted from Weinstein, Sandman, Blalock, 2008) (11, 26, 27). Positioning the traits on this model helps illuminate where each may be with respect to adopting recommended behaviors, which can inform health risk messaging strategies. We place the Protectors trait in “Decided to Act,” the Unengaged in “Unengaged by the Issue,” and the Cautious, Proactive, and Susceptible traits in “Deciding About the Issue.” We also make wildfire smoke health risk messaging recommendations for these traits anchored in a factor important to each trait's perspective profile.

Insights about the perspectives on smoke as a health risk can inform risk communication programs by identifying and addressing the information needs and potential barriers to action. Models of health behaviors, such as the Health Belief Model, Theory of Reasoned Action, and the Theory of Planned Behavior (11, 27–29), suggest an individual's attitudes and perspectives play a role in the adoption of a new health behavior. These models are useful for considering public health risk communication about wildfire smoke, because the goal of the messaging is convincing the intended audience to adopt a recommended behavior that will reduce their risk of adverse outcomes associated with exposure. Ordinarily when health risk communicators are speaking to an audience, many of the audience's demographic characteristics are readily available while the audience's perspectives on the issue are not. When perspectives and demographics do not overlap, it is important that the two are not conflated in thinking about intended audience. The challenge with wildfire smoke is to figure out what the attitudes and perspectives might be across a range of audiences such that public health risk communication can be developed to speak to those perspectives. In a related topic, Leiserowitz et al. (29) provide insights into public engagement efforts related to climate change using segmentation analysis to understand variation in perspectives on the issue of global warming. Their work identified six unique audiences within the broader American public that may respond to engagement efforts related to global warming differently based on their attitudes and perspectives on this issue.

Despite a unique opportunity to provide insight about perspectives shared across recipients of health risk communication strategies, this study has limitations that affect the interpretation and application of these findings. Smoke Sense participants have a different profile than the broader population which may include individuals not affected by smoke and individuals who are not likely to use smartphone apps. Therefore, our findings are not expected to be generalizable to the broader US population. The engagement data has revealed that the primary form of engagement among participants was information seeking through accessing maps and air quality statistics rather than providing information as citizen scientists. As such, most Smoke Sense users may represent an audience that is more likely to receive information through app-based channels—in other words, the type of user that app-based health risk communication strategy would aim to reach. It is also expected that additional personality traits are not included in the data, e.g., measures of willingness to provide data through the app, and these may account for trait attributions. This is a limitation of assessing traits based on a small number of questions. The app also provides an opportunity to iteratively update the reporting instrument as the initiative grows its membership. Each study season provides an opportunity to reflect and revise the reporting instrument based on collective gain in knowledge. For example, based on feedback (16) the Smoke Sense app was translated to Spanish to increase accessibility among Latino Americans. As the initiative grows, we expect that the sample representativeness may increase in all demographic groups.

It is important to consider engagement in app-based health risk communication programs because this may soon become the normal platform for communicating and interacting with public stakeholders and audiences. Compared with other forms of risk communication like fliers, brochures, or advertisements, smart phones provide a platform for immediate, on-demand, and personalized delivery of risk and risk management information. People interact with their mobile devices in an intimate way, using them for communication and for pleasure. These devices are used to conduct business, handle financial matters, get information, and communicate with healthcare providers and receive care, and mobile devices are increasingly being used for health research (30, 31). According to a 2019 Pew Research Center report, approximately 96% of Americans have a cellphone, and 81% have a smartphone, a figure which continues to increase even among older adults aged 65+, and the Deloitte 2018 Global Mobile Consumer Survey: US Edition reports Americans view their smartphones an average of 52 looks per day (32). As smartphones are becoming ubiquitous, public and environmental health programs are beginning to leverage apps as platforms for services like health risk communication. It is therefore not a surprise therefore that Smoke Sense and similar efforts have been attracting strong interest among those affected by smoke (17). However, it is equally important to understand how individuals engage with health risk messaging delivered through mobile apps.

Risk communication theory and practitioner guidelines emphasize the importance of understanding the intended audience in developing communication and outreach programs (7, 8, 11, 13). However, in the context of smoke—the intended audience may be spread across long geographic distances—making particularly challenging the task of health risk messaging that accounts for the mental models and needs of the range of individuals comprising the intended audience for that messaging. Overall, findings reveal a range of perspective traits that differ on health status, perceptions of smoke as a health risk, and information needs, and who engage differently with the Smoke Sense app depending on perspectives about smoke as a health risk. Additionally, information from trusted sources is an important factor for individuals who are affected by wildfire smoke, and future research that examines trusted sources of smoke communication is needed. App-based citizen science health risk messaging is an innovative tool to use for sharing information and health risk messages about wildfire smoke, as well as collecting data from individuals about their smoke experiences, perspectives, and perceptions on health risk messaging. Future directions for research include exploring and examining these data from a behavioral economics lens as well as, additional factors that may influence group cluster membership and engagement, including individual characteristics and objective smoke measurements.

## Conclusions

By examining responses among Smoke Sense participants, we observed heterogeneity among individuals' perspectives on the issue of wildfire smoke as a health risk. Health conditions, risk perception, resources, information needs, self-efficacy, and receptiveness to health risk communication all played a role in differentiating five traits. Individuals with different perspective traits also engaged with the app differently. For some, the app was an information resource while individuals with Susceptible and Cautious traits tended to engage more deeply by learning and contributing to collective knowledge as a reporter. Perspective traits also mapped to different positions along the stage of change in the Precaution Adoption Process Model. When taken together the results of this analysis indicate that perspectives on this issue define individuals' progression toward a desired action and that progression may be influenced by issue engagement needs. Different traits have a different need to engage with the issue in order to motivate change. What this means for public health risk communication is that a range of messaging and engagement level approaches may be needed consider the full range of potential audiences.

## Data Availability Statement

The dataset for this study can be found in the U.S. EPA ScienceHub Environmental Dataset Gateway https://edg.epa.gov/metadata/catalog/search/resource/details.page?uuid=https://doi.org/10.23719/1518408.

## Ethics Statement

The studies involving human participants were reviewed and approved by University of North Carolina Chapel Hill Institutional Review Board. Written informed consent for participation was not required for this study in accordance with the national legislation and the institutional requirements.

## Author Contributions

All authors contributed significantly to the development of this manuscript. AR and MH conceptualized the research question, drafted, and revised the manuscript with equal contribution. MH and LW conducted the analysis. AR and BH oversaw the analysis. SP contributed to formulation of a scientific question and in drafting the manuscript.

## Conflict of Interest

The authors declare that the research was conducted in the absence of any commercial or financial relationships that could be construed as a potential conflict of interest.
