# Supplementary material for: Knowing Your Audience: A Typology of Smoke Sense Participants to Inform Wildfire Smoke Health Risk Communication
Source: Front Public Health. 2020 May 5;8:143. doi: 10.3389/fpubh.2020.00143 (PMC7214918; doi:10.3389/fpubh.2020.00143)
Supplement: Supplementary file 1 [file Data_Sheet_1.docx]

Supplementary Material

# Supplementary Data

All data and meta data used in this study can be found in the U.S. EPA ScienceHub Environmental Dataset Gateway DOI:10.23719/1518408 <https://sciencehub.epa.gov/sciencehub/datasets/2429>

# Supplementary Figures and Tables

Supplementary Table 1: Profile Questions Included in the Smoke Sense Mobile App

| **User Profile Question** | **Response Options** |
| --- | --- |
| 1. Enter your zip code: | 1. [*Type in zip code or use location finding feature*] |
| 1. What is your gender? | 1. Female 2. Male 3. Other |
| 1. Your age? | 1. 18—29 2. 30—39 3. 40—49 4. 50—64 5. 65+ |
| 1. What race/ethnicity best describes you? (Select all that apply) | 1. White 2. African-American/Black 3. Asian/Pacific Islander 4. Native American 5. Hispanic/Latino 6. Other |
| 1. What is your highest education level? | 1. High school degree, GED, or less 2. Technical school, trade or vocational training, or associate degree 3. Bachelors, masters, doctorate, or professional degree |
| 1. Would you say your own health, in general is: | 1. Excellent 2. Very Good 3. Good 4. Fair 5. Poor |
| 1. Select conditions that currently affect your health: (Select all that apply) | 1. Asthma 2. Chronic Obstructive Pulmonary Disease (COPD) 3. Other respiratory disease 4. Hypertension or high blood pressure 5. Other heart disease 6. Type II diabetes, metabolic syndrome, or obesity 7. Allergies related to the upper respiratory tract, eyes, and ears 8. Other chronic disease 9. None of the above |
| 1. Do you commonly experience any of these symptoms? (Select all that apply) | 1. Coughing, trouble breathing, wheezing, asthma attacks, or similar 2. High blood pressure 3. Chest pain or tightness, rapid or irregular heartbeat, or similar 4. Stinging eyes, scratchy throat, or similar 5. Runny or stuffy nose, irritated sinuses, or similar 6. Tiredness, headaches, or similar 7. Trouble sleeping 8. None of the above |
| 1. Which of the following do you use or have readily available to use? (Select all that apply) | 1. A HEPA (high efficiency particulate air) room purifier 2. A car with recirculate mode for the ventilation system 3. Single room air conditioner(s) 4. Whole house (central) air conditioning 5. Work place air conditioning 6. An N95 (or similar) respirator mask 7. Protective gear such as plastic gloves and goggles 8. Access to Air Quality Index levels and associated health related warnings 9. None of the above |
| 1. On average, when you are outside, how active are you? | 1. Not Very Active 2. Mild (walking, standing) 3. Moderate (regular jog, gardening) 4. Very Active (run, bike daily, work outdoors) |
| 1. Please rate your level of agreement:   Smoke from wildfires is a common occurrence where I live. | 1. Strongly agree 2. Somewhat agree 3. Neither agree nor disagree 4. Somewhat disagree 5. Strongly disagree |
| 1. Please rate your level of agreement:   A few hours of wildfire smoke in the air can impact my health. | 1. Strongly agree 2. Somewhat agree 3. Neither agree nor disagree 4. Somewhat disagree 5. Strongly disagree |
| 1. Please rate your level of agreement:   It is possible for me to reduce my wildfire smoke exposure. | 1. Strongly agree 2. Somewhat agree 3. Neither agree nor disagree 4. Somewhat disagree 5. Strongly disagree |
| 1. Please rate your level of agreement:   Information alerts are likely to help me reduce my exposure to wildfire smoke. | 1. Strongly agree 2. Somewhat agree 3. Neither agree nor disagree 4. Somewhat disagree 5. Strongly disagree |
| 1. Information alerts are likely to help me reduce my exposure to wildfire smoke. | 1. whether smoke impacts my health 2. whether specific measures will help my health 3. whether the measure was recommended by a trusted source 4. the effort required for a specific measure 5. the monetary costs of a specific measure 6. I would not consider reducing exposure 7. I don’t need additional information before I reduce exposure |

**Supplementary Table 2: Distribution of responses to measures of perspectives by clusters.**

|  | **Protectors** | **Cautious** | **Proactive** | **Susceptible** | **Unengaged** | **Sample** | Chi-  Square |
| --- | --- | --- | --- | --- | --- | --- | --- |
|  | n=1197 | n=1293 | n=1421 | n=502 | n=605 | n=5018 |  |
|  | **Percent of Responses** | | | | | |  |
| **Personal Health Assessment:** “Would you say your own health, in general is:” | | | | | | | ******** |
| Excellent | *15.54* | *19.49* | **33.29** | *9.36* | **38.02** | 23.67 |  |
| Very Good | *38.85* | **43.85** | **49.40** | *32.07* | *37.85* | 42.33 |  |
| Good | **34.50** | **29.78** | *15.97* | **34.86** | *19.50* | 26.27 |  |
| Fair | **9.19** | *6.26* | *1.27* | **18.13** | *2.98* | 6.34 |  |
| Poor | **1.92** | *0.62* | *0.07* | **5.58** | **1.65** | 1.39 |  |
| **Personal Health Current Symptoms:** “Do you commonly experience any of these symptoms? (Select all that apply):” | | | | | | | |
| Coughing, trouble breathing, wheezing, asthma attacks, or similar | **32.79** | **21.24** | NA | **52.50** | *1.01* | 18.29 | **** |
| High blood pressure, chest pain or tightness, rapid or irregular heartbeat, or similar | **14.31** | **9.17** | NA | **28.54** | *0.51* | 8.53 | **** |
| Stinging eyes, scratchy throat, or similar | **41.74** | **23.81** | *0.07* | **72.85** | *0.67* | 23.04 | **** |
| Runny or stuffy nose, irritated sinuses, or similar | **67.21** | **41.67** | *0.43* | **90.22** | *0.17* | 35.19 | **** |
| Tiredness, headaches, or similar | **52.47** | **36.20** | *0.07* | **77.45** | *0.67* | 29.08 | **** |
| None of the above | *2.47* | *13.44* | **99.43** | NA | **97.14** | 43.18 | **** |
| **Experience with wildfire smoke:** “Smoke from wildfires is a common occurrence where I live.” | | | | | | | **** |
| Strongly agree | **40.43** | **41.53** | *34.48* | **41.43** | *29.59* | 37.82 |  |
| Somewhat agree | *33.67* | **36.04** | **39.13** | *31.27* | *29.92* | 35.13 |  |
| Neither agree nor disagree | **12.53** | *9.98* | *11.68* | *12.35* | **18.68** | 12.36 |  |
| Somewhat disagree | *6.93* | **7.35** | **7.53** | *6.97* | **7.27** | 7.25 |  |
| Strongly disagree | *6.43* | *5.10* | *7.18* | **7.97** | **14.55** | 7.43 |  |
| **Risk Perception:** “A few hours of wildfire smoke in the air can impact my health.” | | | | | | | **** |
| Strongly agree | **61.07** | **59.01** | *51.16* | **69.32** | *39.01* | 55.90 |  |
| Somewhat agree | *26.90* | **30.24** | **30.19** | *22.51* | *26.61* | 28.22 |  |
| Neither agree nor disagree | *8.77* | *8.20* | **13.23** | *6.97* | **21.98** | 11.30 |  |
| Somewhat disagree | *1.75* | *1.93* | **4.15** | *1.00* | **5.29** | 2.83 |  |
| Strongly disagree | *1.50* | *0.62* | *1.27* | *0.20* | **7.11** | 1.75 |  |
| **Self-Efficacy to Reduce Exposure:** “It is possible for me to reduce my wildfire smoke exposure.” | | | | | | | **** |
| Strongly agree | *31.75* | **32.64** | **36.17** | *26.29* | *30.41* | 32.52 |  |
| Somewhat agree | *41.77* | **44.39** | **42.65** | **47.61** | *35.21* | 42.49 |  |
| Neither agree nor disagree | **15.71** | *13.69* | *12.88* | *14.74* | **22.31** | 15.09 |  |
| Somewhat disagree | **7.69** | **6.88** | *6.12* | **7.97** | *5.12* | 6.76 |  |
| Strongly disagree | *3.09* | *2.40* | *2.18* | **3.39** | **6.94** | 3.15 |  |
| **Access to Exposure-Reducing Resources:** “Which of the following do you use or have readily available to use? (Select all that apply)” | | | | | | | |
| A HEPA (high efficiency particulate air) room purifier | **33.95** | **35.31** | *27.54* | *27.40* | *15.03* | 29.25 | **** |
| A car with recirculate mode for the ventilation system | *57.90* | *57.19* | **76.20** | **70.80** | *7.35* | 57.59 | **** |
| Single room air conditioner(s) | *9.75* | **14.06** | *12.36* | **28.60** | *0.67* | 12.28 | **** |
| Whole house (central) air conditioning | **51.26** | *42.97* | **54.73** | **56.60** | *24.37* | 46.99 | **** |
| Work place air conditioning | *34.20* | **37.36** | **50.35** | *30.80* | *9.02* | 35.89 | **** |
| An N95 (or similar) respirator mask | **37.82** | **45.58** | **46.89** | *28.00* | *7.51* | 37.39 | **** |
| Protective gear e.g. plastic gloves and goggles | *15.46* | **20.30** | **27.90** | *11.80* | *2.34* | 18.11 | **** |
| Access to AQI and AQ health related warnings | *41.51* | **44.15** | **58.69** | *37.00* | *7.51* | 42.13 | **** |
| None of the above | *10.25* | *9.00* | NA | *3.20* | **51.25** | 11.14 | **** |
| **Health Messaging Receptiveness:** “Information alerts are likely to help me reduce my exposure to wildfire smoke.” | | | | | | | **** |
| Strongly agree | *52.21* | **59.94** | **55.52** | **59.16** | *41.82* | 54.58 |  |
| Somewhat agree | **33.33** | *31.25* | **32.65** | *30.48* | *28.76* | 31.77 |  |
| Neither agree nor disagree | **10.78** | *6.96* | *9.36* | *8.57* | **20.99** | 10.40 |  |
| Somewhat disagree | **2.76** | *1.47* | *1.41* | *1.00* | **3.14** | 1.91 |  |
| Strongly disagree | *0.92* | *0.39* | *1.06* | *0.80* | **5.29** | 1.34 |  |
| **Information Needs:** “Before considering reducing wildfire smoke exposure, I need more information on: (Select all that apply)” | | | | | | | |
| Whether smoke impacts my health | *14.04* | **41.92** | *29.92* | **46.52** | *25.63* | 30.59 | **** |
| Whether specific measures will help my health | *9.51* | **60.17** | **45.92** | **77.05** | *3.85* | 39.61 | **** |
| Whether the measure was recommended by a trusted source | *10.99* | **57.83** | **39.77** | **65.57** | *10.02* | 37.08 | **** |
| The effort required for a specific measure | *8.40* | **35.17** | **25.31** | **43.44** | *8.48* | 23.92 | **** |
| The monetary costs of a specific measure | *4.34* | **31.50** | *19.38* | **39.55** | *5.39* | 19.56 | **** |
| I would not consider reducing exposure | *1.11* | **2.25** | *0.77* | **2.87** | **4.43** | 1.87 | **** |
| I don't need additional information before I reduce exposure | **68.14** | *7.58* | *33.00* | *5.33* | **64.16** | 35.23 | **** |
| Italics denote cluster values < sample mean  Bold denotes cluster values > sample mean  ****p-value < 0.001 | | | | | | | |

**Supplementary Table 3: Distribution of demographic characteristics by cluster and overall, given as sample percents. Note some questions have multiple response selection option so that responses may not add up to 100%.**

| **Profile Question** | **Protectors** | **Cautious** | **Proactive** | **Susceptible** | **Unengaged** | **Sample** |
| --- | --- | --- | --- | --- | --- | --- |
| **N** | 1197 | 1293 | 1421 | 502 | 605 | 5018 |
| **What race/ethnicity best describes you? (Select all that apply)** | | | | | | |
| White | 77.4 | 74.7 | 79.5 | 78.1 | 68.8 | 76.3 |
| African American/Black | 1.8 | 2.6 | 1.8 | 2.2 | 2.8 | 2.2 |
| Asian/Pacific Islander | 7.9 | 9.7 | 8.2 | 9.0 | 8.9 | 8.7 |
| Native American | 1.6 | 1.7 | 1.2 | 3.4 | 2.5 | 1.8 |
| Hispanic/Latino | 8.0 | 7.8 | 7.0 | 7.6 | 9.8 | 7.8 |
| Other | 7.5 | 6.5 | 5.5 | 6.0 | 11.9 | 7.1 |
| NA | 0.4 | 1.0 | 1.3 | 1.0 | 0.8 | 0.9 |
| **Your age?** | | | | | | |
| 18-29 | 14.4 | 13.8 | 12.2 | 18.5 | 21.7 | 14.9 |
| 30-39 | 20.1 | 22.6 | 24.8 | 17.5 | 19.5 | 21.7 |
| 40-49 | 20.7 | 23.4 | 24.0 | 20.9 | 22.1 | 22.5 |
| 50-64 | 30.3 | 27.5 | 28.3 | 25.3 | 25.1 | 27.9 |
| 65+ | 14.0 | 12.8 | 10.4 | 17.5 | 11.2 | 12.7 |
| NA | 0.5 | NA | 0.4 | 0.2 | 0.3 | 0.3 |
| **Select conditions that currently affect your health: (Select all that apply)** | | | | | | |
| Asthma | 27.7 | 23.8 | 8.0 | 37.3 | 6.6 | 19.5 |
| Chronic Obstructive Pulmonary Disease (COPD) | 3.8 | 1.3 | 0.4 | 4.4 | 0.8 | 1.9 |
| Other respiratory disease | 4.3 | 3.9 | 0.7 | 9.6 | 0.7 | 3.3 |
| Hypertension or high blood pressure | 15.9 | 14.2 | 7.3 | 23.7 | 5.5 | 12.5 |
| Other heart disease | 3.5 | 3.0 | 1.6 | 7.2 | 0.8 | 2.9 |
| Type II diabetes, metabolic syndrome, or obesity | 7.7 | 7.3 | 3.1 | 12.7 | 3.3 | 6.3 |
| Allergies related to the upper respiratory tract, eyes, and ears | 42.6 | 30.4 | 8.9 | 59.8 | 5.3 | 27.1 |
| Other chronic disease | 11.0 | 8.0 | 3.7 | 20.1 | 2.3 | 8.0 |
| None of the above | 30.0 | 35.3 | 71.4 | 17.1 | 77.4 | 47.5 |
| NA | 1.3 | 3.1 | 2.0 | 0.8 | 1.5 | 1.9 |
| **On average, when you are outside, how active are you?** | | | | | | |
| Not Very Active | 5.4 | 5.0 | 2.6 | 7.4 | 6.3 | 4.8 |
| Mild (walking, standing) | 51.1 | 47.8 | 36.7 | 54.0 | 35.7 | 44.6 |
| Moderate (regular jog, gardening) | 31.3 | 31.2 | 40.6 | 26.9 | 28.1 | 33.1 |
| Very Active (run, bike daily, work outdoors) | 12.1 | 15.9 | 20.1 | 11.8 | 29.9 | 17.5 |
| **What is your gender?** | | | | | | |
| Male | 38.8 | 40.8 | 53.1 | 32.9 | 59.8 | 45.3 |
| Female | 59.8 | 57.2 | 45.5 | 65.3 | 35.9 | 52.8 |
| Other | 1.0 | 1.7 | 1.0 | 1.8 | 4.0 | 1.6 |
| NA | 0.3 | 0.3 | 0.4 | NA | 0.3 | 0.3 |
| **What is your highest education level?** | | | | | | |
| High school degree, GED, or less | 15.5 | 10.8 | 7.9 | 16.1 | 19.2 | 12.6 |
| Technical school, trade or vocational training, or associate degree | 18.7 | 17.9 | 11.7 | 21.3 | 13.2 | 16.1 |
| Bachelors, masters, doctorate, or professional degree | 63.7 | 69.9 | 79.0 | 61.0 | 64.8 | 69.5 |
| NA | 2.2 | 1.5 | 1.4 | 1.6 | 2.8 | 1.8 |

**Supplementary Table** **4: Probability of cluster membership for each demographic type. Probabilities are estimated based on multinomial model of cluster belonging given the demographic variable. See Supplemental Materials Figures 2 through 7 for graphical illustration.**

|  |  | **95% C.I.** | |
| --- | --- | --- | --- |
| **On average, when you are outside, how active are you?** | **Probability** | **Lower** | **Upper** |
| **Response** |  |  |  |
| **Protectors** | | | |
| Not Very Active | 0.269 | 0.217 | 0.328 |
| Mild (walking, standing) | 0.273 | 0.255 | 0.292 |
| Moderate (regular jog, gardening) | 0.226 | 0.206 | 0.247 |
| Very Active (run, bike daily, work outdoors) | 0.166 | 0.142 | 0.192 |
| **Cautious** | | | |
| Not Very Active | 0.269 | 0.217 | 0.328 |
| Mild (walking, standing) | 0.276 | 0.258 | 0.295 |
| Moderate (regular jog, gardening) | 0.243 | 0.223 | 0.264 |
| Very Active (run, bike daily, work outdoors) | 0.235 | 0.208 | 0.264 |
| **Proactive** | | | |
| Not Very Active | 0.153 | 0.113 | 0.204 |
| Mild (walking, standing) | 0.233 | 0.216 | 0.251 |
| Moderate (regular jog, gardening) | 0.347 | 0.325 | 0.371 |
| Very Active (run, bike daily, work outdoors) | 0.325 | 0.295 | 0.357 |
| **Susceptible** | | | |
| Not Very Active | 0.153 | 0.113 | 0.204 |
| Mild (walking, standing) | 0.121 | 0.108 | 0.135 |
| Moderate (regular jog, gardening) | 0.081 | 0.069 | 0.095 |
| Very Active (run, bike daily, work outdoors) | 0.067 | 0.053 | 0.086 |
| **Unengaged** | | | |
| Not Very Active | 0.157 | 0.116 | 0.208 |
| Mild (walking, standing) | 0.096 | 0.085 | 0.109 |
| Moderate (regular jog, gardening) | 0.102 | 0.089 | 0.118 |
| Very Active (run, bike daily, work outdoors) | 0.207 | 0.181 | 0.235 |
|  | | | |
| **What is your gender?** | | **95% C.I.** | |
| **Response** | **Probability** | **Lower** | **Upper** |
| **Protectors** | | | |
| Male | 0.204 | 0.188 | 0.222 |
| Female | 0.270 | 0.254 | 0.288 |
| Other Gender | 0.148 | 0.086 | 0.243 |
| **Cautious** | | | |
| Male | 0.232 | 0.215 | 0.250 |
| Female | 0.280 | 0.263 | 0.297 |
| Other Gender | 0.272 | 0.186 | 0.378 |
| **Proactive** | | | |
| Male | 0.332 | 0.313 | 0.352 |
| Female | 0.244 | 0.228 | 0.261 |
| Other Gender | 0.173 | 0.105 | 0.271 |
| **Susceptible** | | | |
| Male | 0.073 | 0.063 | 0.084 |
| Female | 0.124 | 0.112 | 0.137 |
| Other Gender | 0.111 | 0.059 | 0.200 |
| **Unengaged** | | | |
| Male | 0.159 | 0.145 | 0.175 |
| Female | 0.082 | 0.072 | 0.093 |
| Other Gender | 0.296 | 0.207 | 0.404 |
|  | | | |
| **What is your highest education level?** | | **95% C.I.** | |
| **Response** | **Probability** | **Lower** | **Upper** |
| **Protectors** | | | |
| High school degree, GED, or less | 0.292 | 0.258 | 0.329 |
| Bachelors, masters, doctorate, or professional degree | 0.219 | 0.205 | 0.233 |
| Technical school, trade or vocational training, or associate degree | 0.277 | 0.247 | 0.309 |
| **Cautious** | | | |
| High school degree, GED, or less | 0.220 | 0.189 | 0.254 |
| Bachelors, masters, doctorate, or professional degree | 0.259 | 0.245 | 0.274 |
| Technical school, trade or vocational training, or associate degree | 0.286 | 0.256 | 0.318 |
| **Proactive** | | | |
| High school degree, GED, or less | 0.177 | 0.149 | 0.209 |
| Bachelors, masters, doctorate, or professional degree | 0.322 | 0.307 | 0.338 |
| Technical school, trade or vocational training, or associate degree | 0.205 | 0.179 | 0.235 |
| **Susceptible** | | | |
| High school degree, GED, or less | 0.128 | 0.104 | 0.156 |
| Bachelors, masters, doctorate, or professional degree | 0.088 | 0.079 | 0.098 |
| Technical school, trade or vocational training, or associate degree | 0.132 | 0.111 | 0.158 |
| **Unengaged** | | | |
| High school degree, GED, or less | 0.183 | 0.155 | 0.215 |
| Bachelors, masters, doctorate, or professional degree | 0.112 | 0.102 | 0.123 |
| Technical school, trade or vocational training, or associate degree | 0.099 | 0.080 | 0.122 |
|  | | | |
| **Your age?** |  | **95% C.I.** | |
| **Response** | **Probability** | **Lower** | **Upper** |
| **Protectors** | | | |
| 18-29 | 0.230 | 0.201 | 0.262 |
| 30-39 | 0.220 | 0.197 | 0.246 |
| 40-49 | 0.219 | 0.196 | 0.244 |
| 50-64 | 0.259 | 0.237 | 0.283 |
| 65+ | 0.264 | 0.231 | 0.299 |
| **Cautious** | | | |
| 18-29 | 0.238 | 0.209 | 0.270 |
| 30-39 | 0.268 | 0.242 | 0.295 |
| 40-49 | 0.268 | 0.243 | 0.294 |
| 50-64 | 0.254 | 0.232 | 0.277 |
| 65+ | 0.259 | 0.226 | 0.294 |
| **Proactive** | | | |
| 18-29 | 0.232 | 0.203 | 0.263 |
| 30-39 | 0.323 | 0.296 | 0.351 |
| 40-49 | 0.302 | 0.275 | 0.329 |
| 50-64 | 0.287 | 0.264 | 0.312 |
| 65+ | 0.232 | 0.201 | 0.267 |
| **Susceptible** | | | |
| 18-29 | 0.124 | 0.103 | 0.150 |
| 30-39 | 0.081 | 0.066 | 0.098 |
| 40-49 | 0.093 | 0.077 | 0.111 |
| 50-64 | 0.091 | 0.077 | 0.107 |
| 65+ | 0.138 | 0.113 | 0.167 |
| **Unengaged** | | | |
| 18-29 | 0.175 | 0.150 | 0.204 |
| 30-39 | 0.108 | 0.091 | 0.128 |
| 40-49 | 0.118 | 0.101 | 0.139 |
| 50-64 | 0.109 | 0.093 | 0.126 |
| 65+ | 0.107 | 0.085 | 0.133 |
|  | | | |
| **Select conditions that currently affect your health: (Select all that apply)** |  | **95% C.I.** | |
| **Response** | **Probability** | **Lower** | **Upper** |
| **Protectors** | | | |
| None of the above | 0.151 | 0.137 | 0.166 |
| Allergies related to the upper respiratory tract, eyes, and ears | 0.345 | 0.310 | 0.382 |
| Asthma | 0.336 | 0.307 | 0.367 |
| Chronic Obstructive Pulmonary Disease (COPD) | 0.458 | 0.336 | 0.585 |
| Hypertension or high blood pressure | 0.277 | 0.236 | 0.322 |
| Other chronic disease | 0.279 | 0.210 | 0.361 |
| Other heart disease | 0.352 | 0.237 | 0.487 |
| Other respiratory disease | 0.312 | 0.226 | 0.413 |
| Type II diabetes, metabolic syndrome, or obesity | 0.254 | 0.185 | 0.339 |
| **Cautious** | | | |
| None of the above | 0.191 | 0.176 | 0.208 |
| Allergies related to the upper respiratory tract, eyes, and ears | 0.325 | 0.290 | 0.361 |
| Asthma | 0.316 | 0.287 | 0.346 |
| Chronic Obstructive Pulmonary Disease (COPD) | 0.204 | 0.119 | 0.325 |
| Hypertension or high blood pressure | 0.298 | 0.256 | 0.344 |
| Other chronic disease | 0.301 | 0.230 | 0.384 |
| Other heart disease | 0.240 | 0.145 | 0.371 |
| Other respiratory disease | 0.355 | 0.264 | 0.457 |
| Type II diabetes, metabolic syndrome, or obesity | 0.361 | 0.280 | 0.449 |
| **Proactive** | | | |
| None of the above | 0.426 | 0.406 | 0.445 |
| Allergies related to the upper respiratory tract, eyes, and ears | 0.140 | 0.115 | 0.168 |
| Asthma | 0.115 | 0.096 | 0.136 |
| Chronic Obstructive Pulmonary Disease (COPD) | 0.085 | 0.036 | 0.188 |
| Hypertension or high blood pressure | 0.217 | 0.180 | 0.259 |
| Other chronic disease | 0.235 | 0.172 | 0.314 |
| Other heart disease | 0.222 | 0.131 | 0.352 |
| Other respiratory disease | 0.086 | 0.044 | 0.163 |
| Type II diabetes, metabolic syndrome, or obesity | 0.197 | 0.136 | 0.277 |
| **Susceptible** | | | |
| None of the above | 0.036 | 0.029 | 0.044 |
| Allergies related to the upper respiratory tract, eyes, and ears | 0.154 | 0.129 | 0.183 |
| Asthma | 0.192 | 0.168 | 0.218 |
| Chronic Obstructive Pulmonary Disease (COPD) | 0.169 | 0.094 | 0.287 |
| Hypertension or high blood pressure | 0.136 | 0.106 | 0.172 |
| Other chronic disease | 0.103 | 0.062 | 0.166 |
| Other heart disease | 0.148 | 0.076 | 0.269 |
| Other respiratory disease | 0.215 | 0.143 | 0.310 |
| Type II diabetes, metabolic syndrome, or obesity | 0.090 | 0.051 | 0.156 |
| **Unengaged** | | | |
| None of the above | 0.196 | 0.181 | 0.213 |
| Allergies related to the upper respiratory tract, eyes, and ears | 0.037 | 0.025 | 0.054 |
| Asthma | 0.041 | 0.030 | 0.055 |
| Chronic Obstructive Pulmonary Disease (COPD) | 0.085 | 0.036 | 0.188 |
| Hypertension or high blood pressure | 0.072 | 0.050 | 0.101 |
| Other chronic disease | 0.081 | 0.045 | 0.140 |
| Other heart disease | 0.037 | 0.009 | 0.136 |
| Other respiratory disease | 0.032 | 0.010 | 0.095 |
| Type II diabetes, metabolic syndrome, or obesity | 0.098 | 0.057 | 0.165 |
|  | | | |
| **What race/ethnicity best describes you? (Select all that apply)** |  | **95% C.I.** | |
| **Response** | **Probability** | **Lower** | **Upper** |
| **Protectors** | | | |
| White | 0.242 | 0.229 | 0.256 |
| African-American/Black | 0.200 | 0.130 | 0.295 |
| Asian/Pacific Islander | 0.207 | 0.170 | 0.251 |
| Hispanic/Latino | 0.248 | 0.203 | 0.298 |
| Native American | 0.214 | 0.126 | 0.341 |
| Other Race/Ethnicity | 0.260 | 0.214 | 0.312 |
| **Cautious** | | | |
| White | 0.252 | 0.239 | 0.266 |
| African-American/Black | 0.322 | 0.234 | 0.425 |
| Asian/Pacific Islander | 0.299 | 0.255 | 0.347 |
| Hispanic/Latino | 0.270 | 0.224 | 0.321 |
| Native American | 0.268 | 0.168 | 0.398 |
| Other Race/Ethnicity | 0.237 | 0.193 | 0.288 |
| **Proactive** | | | |
| White | 0.295 | 0.280 | 0.309 |
| African-American/Black | 0.256 | 0.176 | 0.355 |
| Asian/Pacific Islander | 0.268 | 0.226 | 0.314 |
| Hispanic/Latino | 0.238 | 0.195 | 0.288 |
| Native American | 0.161 | 0.086 | 0.281 |
| Other Race/Ethnicity | 0.221 | 0.178 | 0.271 |
| **Susceptible** | | | |
| White | 0.102 | 0.093 | 0.112 |
| African-American/Black | 0.078 | 0.038 | 0.154 |
| Asian/Pacific Islander | 0.105 | 0.078 | 0.140 |
| Hispanic/Latino | 0.091 | 0.064 | 0.128 |
| Native American | 0.179 | 0.099 | 0.301 |
| Other Race/Ethnicity | 0.065 | 0.042 | 0.098 |
| **Unengaged** | | | |
| White | 0.109 | 0.099 | 0.119 |
| African-American/Black | 0.144 | 0.086 | 0.233 |
| Asian/Pacific Islander | 0.121 | 0.092 | 0.157 |
| Hispanic/Latino | 0.154 | 0.118 | 0.197 |
| Native American | 0.179 | 0.099 | 0.301 |
| Other Race/Ethnicity | 0.218 | 0.175 | 0.267 |

**
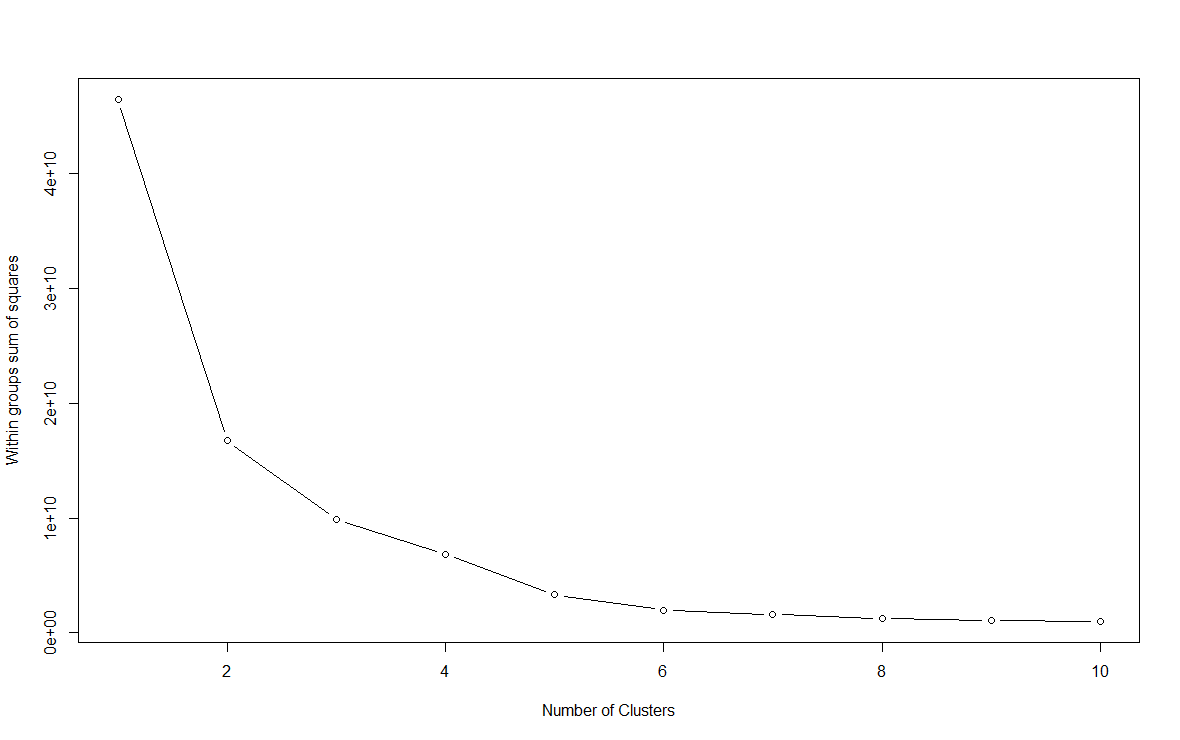
Supp**

**Supplemental Materials Figure 1: 5-Cluster Solution Identified Based on Comparison of Within Case Sum of Squares**


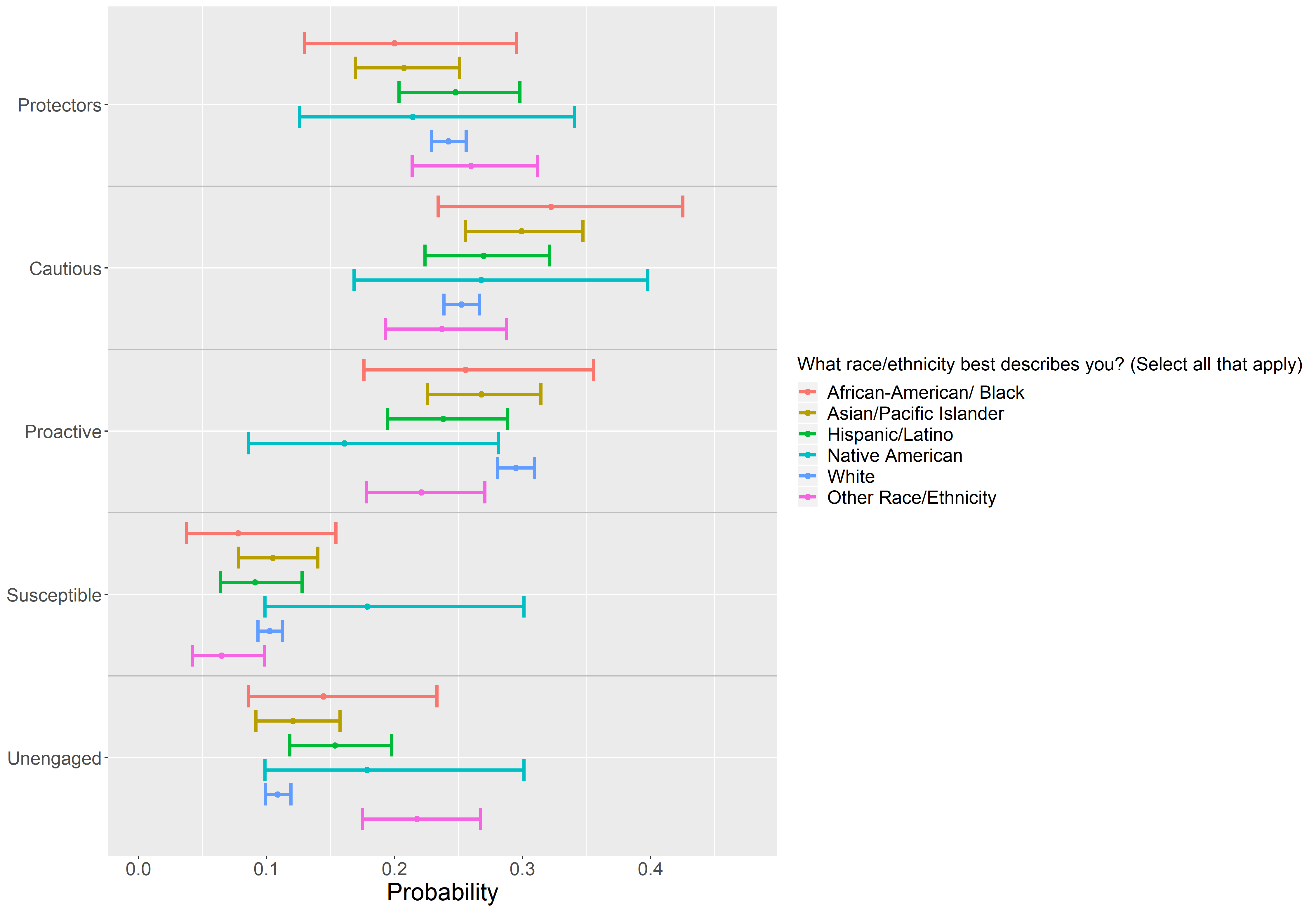


**Supplemental Materials Figure 2: Probability of cluster membership based on race. Probabilities are estimated based on multinomial model of cluster belonging given the demographic variable. Figures correspond to probabilities given in Supplemental Materials Table 4**


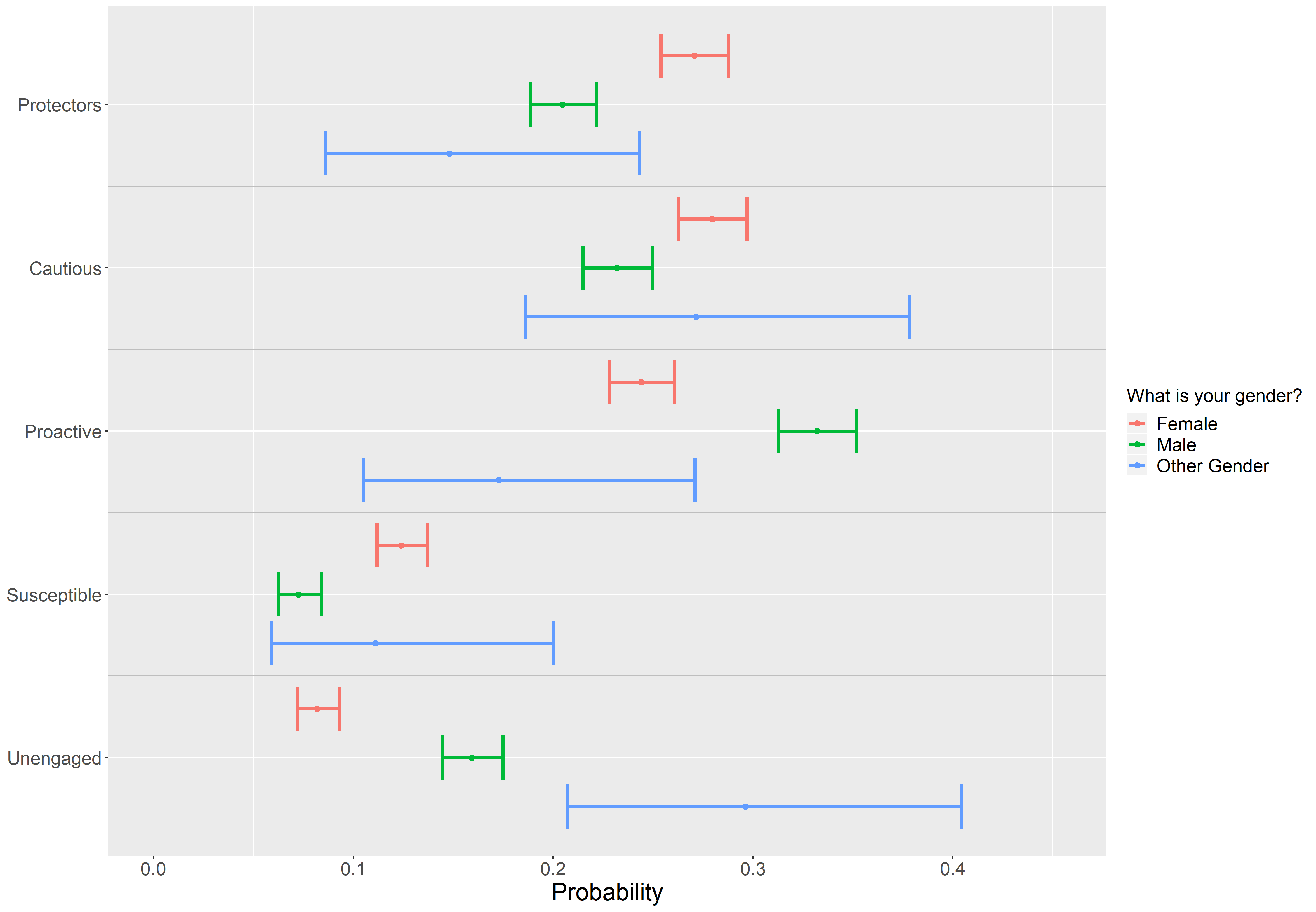
 **Supplemental Materials Figure 3: Probability of cluster membership based on gender. Probabilities are estimated based on multinomial model of cluster belonging given the demographic variable. Figures correspond to probabilities given in Supplemental Materials Table 4**


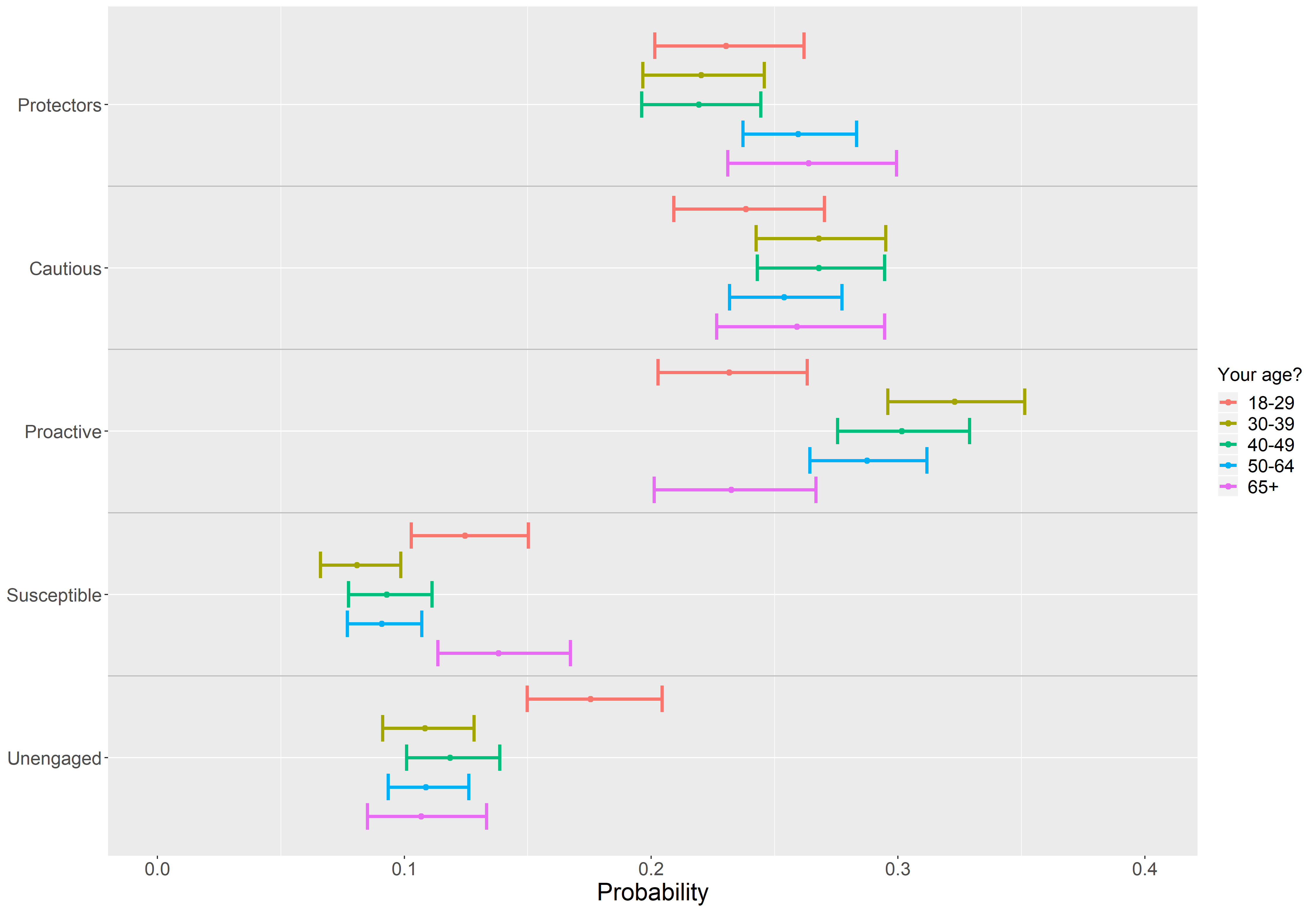


**Supplemental Materials Figure 4: Probability of cluster membership based on age. Probabilities are estimated based on multinomial model of cluster belonging given the demographic variable. Figures correspond to probabilities given in Supplemental Materials Table 4**


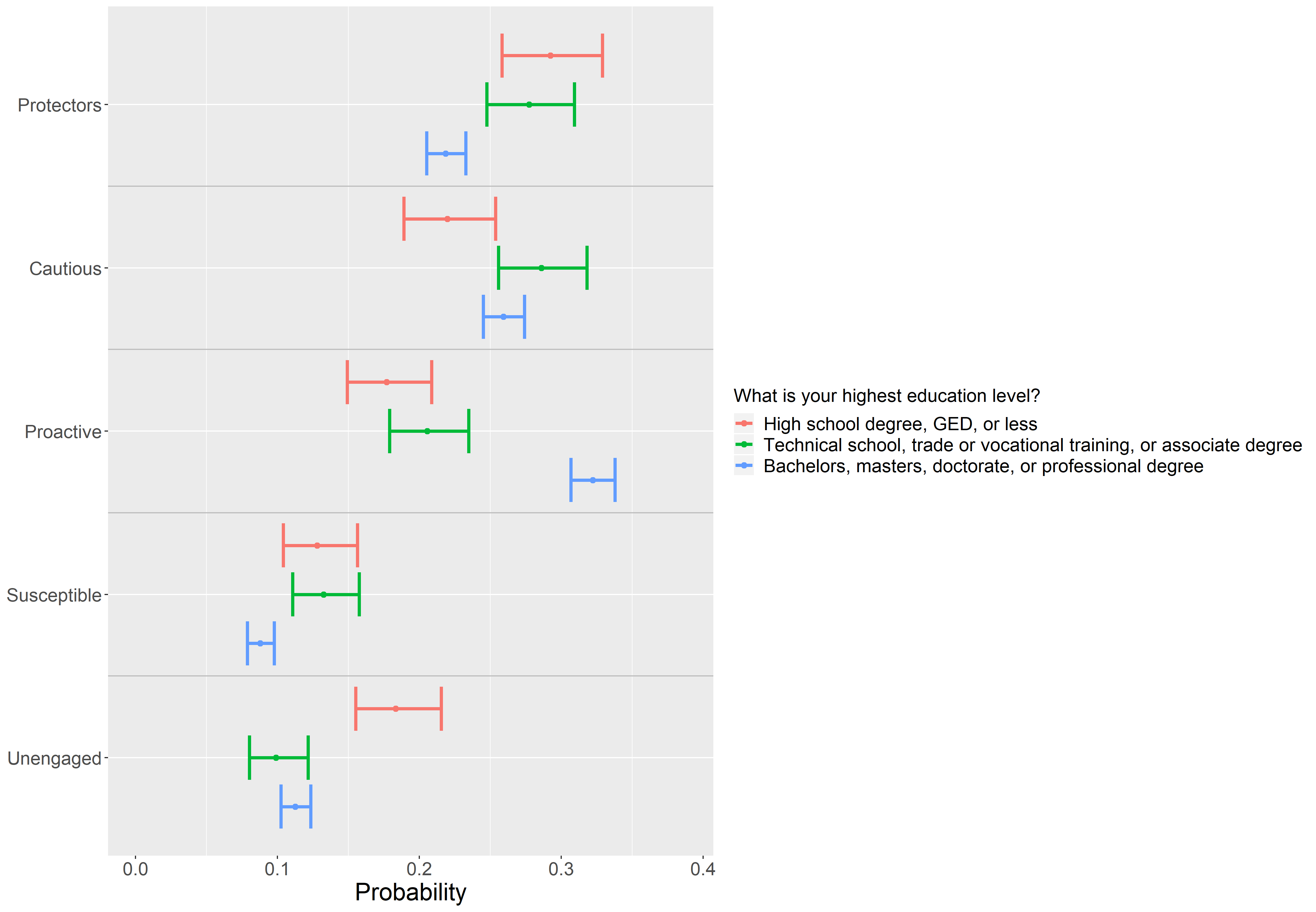


**Supplemental Materials Figure 5: Probability of cluster membership based on highest level of education. Probabilities are estimated based on multinomial model of cluster belonging given the demographic variable. Figures correspond to probabilities given in Supplemental Materials Table 4**


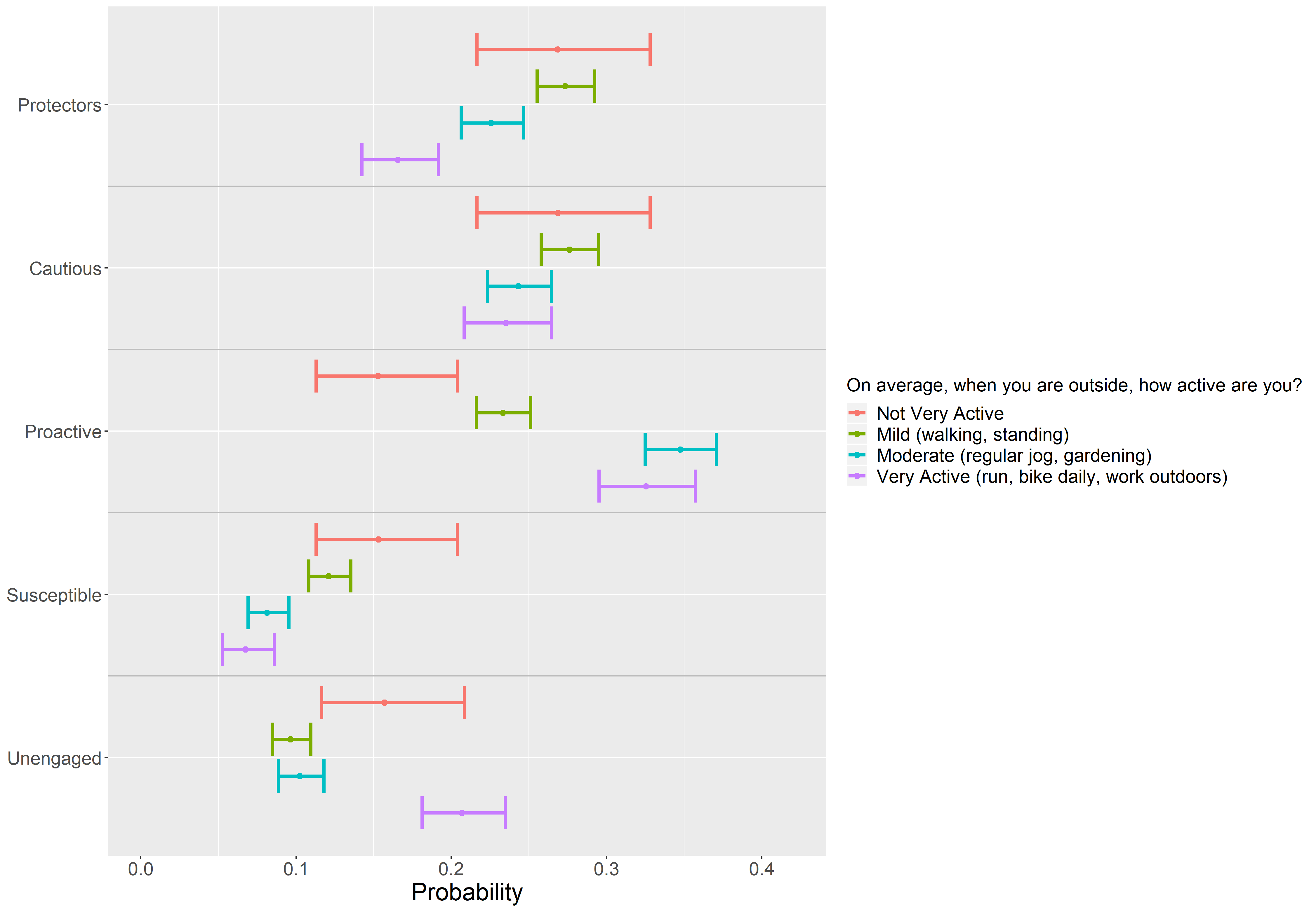
 **Supplemental Materials Figure 6: Probability of cluster membership based on reported average outdoor activity. Probabilities are estimated based on multinomial model of cluster belonging given the demographic variable. Figures correspond to probabilities given in Supplemental Materials Table 4**


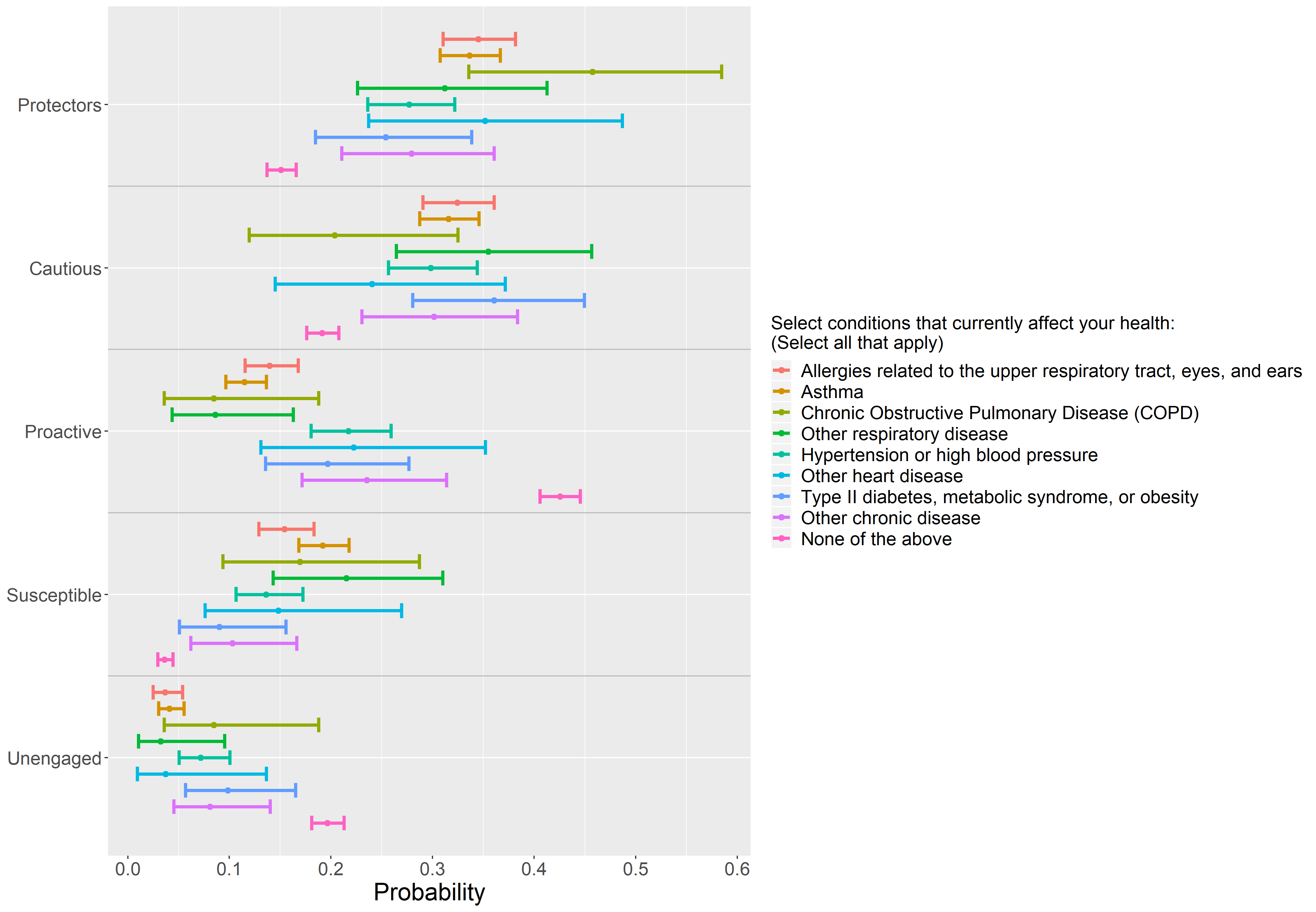
 **Supplemental Materials Figure 7: Probability of cluster membership based on current conditions. Probabilities are estimated based on multinomial model of cluster belonging given the demographic variable. Figures correspond to probabilities given in Supplemental Materials Table 4**
